# The Possibility of Zero Limb-Work Gaits in Sprawled and Parasagittal Quadrupeds: Insights from Linkages of the Industrial Revolution

**DOI:** 10.1093/iob/obaa017

**Published:** 2020-06-04

**Authors:** J R Usherwood

**Affiliations:** Structure and Motion Lab, The Royal Veterinary College, North Mymms, Hatfield, Herts AL9 7TA, UK

## Abstract

Animal legs are diverse, complex, and perform many roles. One defining requirement of legs is to facilitate terrestrial travel with some degree of economy. This could, theoretically, be achieved without loss of mechanical energy if the body could take a continuous horizontal path supported by vertical forces only—effectively a wheel-like translation, and a condition closely approximated by walking tortoises. If this is a potential strategy for zero mechanical work cost among quadrupeds, how might the structure, posture, and diversity of both sprawled and parasagittal legs be interpreted? In order to approach this question, various linkages described during the industrial revolution are considered. Watt’s linkage provides an analogue for sprawled vertebrates that uses diagonal limb support and shows how vertical-axis joints could enable approximately straight-line horizontal translation while demanding minimal mechanical power. An additional vertical-axis joint per leg results in the wall-mounted pull-out monitor arm and would enable translation with zero mechanical work due to weight support, without tipping or toppling. This is consistent with force profiles observed in tortoises. The Peaucellier linkage demonstrates that parasagittal limbs with lateral-axis joints could also achieve the zero-work strategy. Suitably tuned four-bar linkages indicate this is feasibly approximated for flexed, biologically realistic limbs. Where “walking” gaits typically show out of phase fluctuation in center of mass kinetic and gravitational potential energy, and running, hopping or trotting gaits are characterized by in-phase energy fluctuations, the zero limb-work strategy approximated by tortoises would show zero fluctuations in kinetic or potential energy. This highlights that some gaits, perhaps particularly those of animals with sprawled or crouched limbs, do not fit current kinetic gait definitions; an additional gait paradigm, the “zero limb-work strategy” is proposed.

## Synopsis Chinese

动物的腿是复杂多样的, 扮演着许多角色。腿的一个定义性要求是能够经济方便地协助动物在地面上行动。从理论上讲, 如果身体能够在垂直力的支持下沿着一条连续的水平路径移动, 那么就可以在不损失机械能的情况下实现这一点——这非常类似车轮的平移, 与乌龟的行走特性非常相似。如果这是一种四足动物可以实现耗费零机械能的潜在策略, 那么如何解释外展趴卧肢和内收直立肢的结构、姿势和它们的多样性呢?为了解决这一问题, 我们考虑了工业革命期间所描述的各种联动装置。瓦特的联动装置提供了一种类似于四肢爬行的脊椎动物使用对角肢体支撑的方法, 并展示了垂直轴关节如何在需要最小机械动力的情况下实现近似直线的水平移动。每条腿增加一个垂直轴关节使得安装在墙上的拉出监控器手臂可以实现移动, 可以实现在重量的支持方面零机械功的移动, 并且不会倾斜或倾倒。这与在乌龟身上观察到的力分布相一致。Peaucellier 联动装置显示, 具有侧轴关节的内收直立肢也可以实现零功策略。适当调整的四杆机构表明这是可行的近似弯曲、生物学上合理的四肢。“行走”步态一般表现为质心动能和重力势能的非相位涨落, 奔跑、跳跃和小跑步态表现为同相位能量涨落, 而陆龟近似的零肢功策略则表现为动能或势能的零涨落。这突出表明, 某些步态, 特别是那些四肢伸展或蜷曲的动物步态, 不符合当前的动态步态定义;这里我们提出了一种新的步态模式——“零肢体-功策略”。========================================動物的腿是復雜多樣的，扮演著許多角色。腿的壹個定義性要求是能夠經濟方便地協助動物在地面上行動。從理論上講，如果身體能夠在垂直力的支持下沿著壹條連續的水平路徑移動，那麽就可以在不損失機械能的情況下實現這壹點——這非常類似車輪的平移，與烏龜的行走特性非常相似。如果這是壹種四足動物可以實現耗費零機械能的潛在策略，那麽如何解釋外展趴臥肢和內收直立肢的結構、姿勢和它們的多樣性呢?為了解決這壹問題，我們考慮了工業革命期間所描述的各種聯動裝置。瓦特的聯動裝置提供了壹種類似於四肢爬行的脊椎動物使用對角肢體支撐的方法，並展示了垂直軸關節如何在需要最小機械動力的情況下實現近似直線的水平移動。每條腿增加壹個垂直軸關節使得安裝在墻上的拉出監控器手臂可以實現移動，可以實現在重量的支持方面零機械功的移動，並且不會傾斜或傾倒。這與在烏龜身上觀察到的力分布相壹致。Peaucellier 聯動裝置顯示，具有側軸關節的內收直立肢也可以實現零功策略。適當調整的四桿機構表明這是可行的近似彎曲、生物學上合理的四肢。“行走”步態壹般表現為質心動能和重力勢能的非相位漲落，奔跑、跳躍和小跑步態表現為同相位能量漲落，而陸龜近似的零肢功策略則表現為動能或勢能的零漲落。這突出表明，某些步態，特別是那些四肢伸展或蜷曲的動物步態，不符合當前的動態步態定義;這裏我們提出了壹種新的步態模式——“零肢體-功策略”。

## Synopsis German

Gliedmaßen sind vielfältig, komplex und dienen vielen Funktionen. Eine definierende Anforderung an Gliedmaßen ist energie-effiziente Fortbewegung. Dies könnte theoretisch ohne Verlust mechanischer Energie erreicht werden wenn sich der Körperschwerpunkt entlang einer horizintalen Linie bewegt, und daher nur durch vertikale Kräfte unterstützt wird, so wie Fortbewegung auf Rädern, eine Fortbewegunsweise der Schildkröten nahe kommen. Wenn wir annehmen dass diese Strategie es Vierbeiners erlaubt mechanische Arbeit auf null zu reduzieren, kann diese Strategie die Struktur, Haltung und Vielfalt sowohl der gespreizten als auch der parasagittalen Extremitätenführung erklaeren? Wir beantworten diese Frage indem wir verschiedene Gelenkmechanismen aus der industriellen Revolution betrachten. Das Wattsche Parallelogramm entspricht Wirbeltieren mit gespreizten Gliedmaßen im Kreuzgang, und es zeigt wie Gelenke mit vertikaler Achse eine annähernd geradlinige horizontale Fortbewegung ermöglichen und gleichzeitig minimale mechanische Leistung erfordern. Ein zusätzliches Gelenk mit vertikaler Achse pro Gliedmaße führt zu einem Glenkmechanismus der einer ausziehbaren Bieldschirm Wandhalterung gleicht und der Fortbewegung ohne mechanische Arbeit fuer Gewichtsunterstützung und ohne Fallen oder Umfallen ermöglicht. Dies steht im Einklang mit Kraftprofilen, die bei Schildkröten beobachtet werden. Die Peaucellier-Geradführung zeigt, dass parasagittale Gliedmaßen mit Seitenachsengelenken auch der ‚arbeitsfreien Gliedmaßen Stategie” folgen können. Geeignet abgestimmte Gelenkviereckverbindungen zeigen, dass dies für gebogene, biologisch realistische Gliedmaßen möglich ist. Während Gehgänge typischerweise eine Phasenschwankung in der kinetischen Energie des Massenschwerpunkts und des Gravitationspotentials aufweisen und Lauf-, Sprung- und Trabgänge durch gleichphasige Energiefluktuationen gekennzeichnet sind, wäre die kinetischer oder potentieller Energie von gehenden Schildkröten schwankungsfrei. Dies unterstreicht, dass einige Gangarten, insbesondere die von Tieren mit gespreizten oder gebeugten Gliedmaßen, nicht den gegenwärtigen kinetischen Gangdefinitionen entsprechen. Als zusätzliches Gangparadigma wird die „arbeitsfreie Gliedmaßen Stategie “vorgeschlagen.

## Synopsis Japanese

動物の脚は多様かつ複雑で、複数の役割を担っている。脚が果たすべき主要な要件のひとつに、ある程度の経済性を伴って陸上での移動を促進することが挙げられる。もし胴体が垂直方向の力のみで支えられ、鉛直方向への振動なしに水平移動ができれば、理論的には、機械的エネルギを失うことがなく、経済的な歩行が達成される。これは実質的には車輪による並進移動と同等であり、リクガメの歩行で近似できる状態に近い。もしこれが四足歩行性四肢動物の歩行に伴う機械的仕事をゼロにできる潜在的な戦略であるとすれば、肘を張り出す側方型四肢姿勢と肘を体壁に沿わせる下方型四肢姿勢のそれぞれについて、四肢の構造・姿勢・多様性をどのように解釈すればよいのだろうか？この課題に取り組むために、産業革命期に提案された種々のリンケージを検討する。ワット・リンクは、対角線上の脚を体重支持に使う側方型四肢脊椎動物のアナロジーになっており、垂直軸関節によって、ほぼ直線的な並進運動が最小限の機械的パワーでいかに実現されうるかを示している。ここで、各四肢に垂直軸関節を追加すると、これは壁に取り付けた引出式モニタアームに相当し、体重支持に伴う機械的仕事がゼロで、かつ傾きや転倒のない並進移動が可能となる。これは、リクガメに見られる力のプロファイルと合致している。また別のリンケージである Peaucellier リンケージは、左右軸関節をもち矢状面内で脚を動かす方式（下方型四肢）でも仕事ゼロの戦略を達成可能であろうことを示している。さらに、4バーリンケージを適切に調整することで、生物学的により現実的な曲がった四肢でも、仕事ゼロ戦略が近似的に実現可能であることが示唆された。「歩行」ではしばしば、質量中心についての運動エネルギと重力ポテンシャルエネルギの位相にずれが見られる。一方、走行（ランニング）・ホッピング・トロットではエネルギ変動は位相が揃っている。これらに対して、リクガメで近似された「四肢仕事ゼロ戦略」では運動エネルギとポテンシャルエネルギの変動がゼロであると考えられる。このエネルギ変動の明確な違いは、ある種の歩容 (gait)、とりわけ側方型や匍匐型の姿勢をとる四肢動物のそれは、現在の運動学的歩容の定義に合わない可能性を浮き彫りにする。そこで、新たな歩容パラダイム「四肢仕事ゼロ戦略 (zero limb-work strategy)」を提唱する。

## Synopsis Polish

Nogi zwierząt są różnorodne, mają złożoną budowę i pełnią wiele ról. Jednym z podstawowych zastosowań kończyn jest ułatwienie przemieszczanie się naziemnego przy pewnym stopniu oszczędności (energetycznej). Teoretycznie, gdyby ciało (a właściwie jego środek masy), podparte przez pionowe siły reakcji, niczym koło, mogło przemieszczać się jedynie w płaszczyźnie poziomej, chód mógłby odbywać się bez strat energii mechanicznej. Warunek ten jest w przybliżeniu spełniony przez chodzące żółwie. Jeśli przyjmiemy, że minimalizacja kosztu pracy może być strategią u czworonogów, jak można interpretować strukturę, ustawienie i różnorodność zarówno kończyn ułożonych bocznie, jak i bardziej pod ciałem? Aby podejść do tego pytania, w pracy rozważane są różne połączenia i łańcuchy mechaniczne, których opis wywodzi się z rewolucji przemysłowej. Prostowód Watta zapewnia analogię dla kręgowców z kończynami umieszczonymi bocznie, które wykorzystują podparcie kończyn po przekątnej, i wyjaśnia, w jaki sposób połączenia o pionowej osi obrotu mogą umożliwić translację poziomą w przybliżeniu w linii prostej, wymagając minimalnej mocy mechanicznej. Dodatkowe połączenie o pionowej osi obrotu, analogiczne do ramienia montażowego, które pozwala na odsunięcie monitora od ściany, umożliwiłoby translację przy zerowej pracy mechanicznej ze względu na podparcie ciężaru oraz bez przechylania się lub przewracania. Jest to zgodne z profilami siły obserwowanymi u żółwi.Prostowód Peaucellier pokazuje, że kończyny ze stawami o bocznej osi obrotu (zawarte w płaszczyźnie poprzecznej) również umożliwiają osiągnięcie strategii zerowej pracy. Odpowiednio dopasowane czteropunktowe połączenia wskazują, że jest to możliwe w przybliżeniu dla ugiętych, biologicznie realistycznych kończyn. Dla przemieszczania się w postaci typowego chodu, zwykle obserwuje się zmiany energii kinetycznej i potencjalnej środka masy w przeciwfazie, zaś lokomocja w postaci biegu, wieloskoków czy też kłusu charakteryzuje się fluktuacjami energii o zgodnej fazie. Z kolei strategia zerowej pracy kończyn, aproksymowana chodem żółwi, wykazałaby zerowe zmiany energii kinetycznej lub potencjalnej. Podkreśla to, że niektóre rodzaju chodu, być może w szczególności zwierząt z kończynami ułożonymi bardziej z boku ciała lub w ustawieniu zgięciowym, nie pasują do obecnych definicji kinetycznych typów lokomocji. Zaproponowano zatem dodatkowy paradygmat chodu – „strategię zerowej pracy kończyny”.

## Synopsis French

Le squelette appendiculaire des animaux est diversifié, complexe et permet de réaliser de nombreuses fonctions. L”une de ces fonctions principales est de permettre un déplacement efficace en milieu terrestre. En théorie, ce déplacement peut être effectué avec une faible dépense d”énergie mécanique si le corps suit un déplacement horizontal tout en étant soutenu par des forces verticales comme dans le cas d”une translation engendrée par un mouvement de roue: une condition atteinte par les tortues terrestres. Comment interpréter la structure, la posture ainsi que la diversité des membres parasagittaux et « arqués » tout en considérant la stratégie d”atteindre un travail de force nul au niveau des membres chez les organismes quadrupèdes? Pour répondre à cette question, plusieurs mécanismes décrits pendant la révolution industrielle sont considérés. Le mécanisme de Watt peut être utilisé comme une analogie pour les vertébrés avec des membres « arqués » car ils offrent un support diagonal au corps, démontrant ainsi comment les articulations le long d”un axe vertical peuvent permettre une translation horizontale du mouvement tout en demandant un minimum de travail de force. L”analogie qui correspond le mieux à l”ajout d”une articulation supplémentaire le long de l”axe vertical sur chaque membre est celle d”un support mural avec bras articulé, typiquement utilisé pour des écrans. Ce modèle permet une translation avec un travail de force nul grâce au support du poids et à l”absence de basculement, ce qui est également observé chez les tortues terrestres. Le mécanisme de Peaucellier démontre quant à lui que les membres parasagittaux avec des articulations le long d”un axe latéral peuvent également atteindre la condition de travail de force nul. Les mécanismes à quatre barres, avec les paramètres appropriés, fournissent également des approximations biologiquement crédibles de la flexion des membres. Tandis que la marche provoque des fluctuations alternées entre l”énergie cinétique du centre de masse et l”énergie potentielle gravitationnelle alors que la course, le saut ainsi que le trot sont caractérisés par des fluctuations synchronisées, la condition de travail de force nul atteinte par les tortues offre des fluctuations nulles pour l”énergie cinétique et l”énergie potentielle. Ceci démontre que certains modes de locomotions, en particuliers ceux d”animaux avec des membres « arqués » ou une position accroupie, ne correspondent pas aux définitions actuelles. Un nouveau paradigme est donc proposé: « the zero limb-work strategy » (terme français suggéré: « la non-sollicitation des membres »).

## Background and assumptions

There are various demands for effective legged locomotion. Animals require some capacity to accelerate, travel, decelerate, maneuver, climb, and descend, all with adequate control. Such activities are metabolically demanding, so presumably there has been selection acting on the form and use of legs toward economy. The condition for zero mechanical work is the avoidance of any instances of mechanical power; that force and velocity are always perpendicular to each other or that one of them is zero. This generalization accounts for the (theoretically) zero work requirement of rolling wheels, sliding skates, and swinging pendulums. While a variety of biological tissues may absorb and return energy with some degree of elasticity, all do so with hysteresis and energy lost as heat; some relevance of mechanical work and power minimization still applies even for animals with long, elastic tendons.

To what extent can the implications of posture and gait of terrestrial animals be related to minimization of the mechanical work and power demanded from the limbs? And what insight can be found from suitable analogues developed in engineering?

To explore such questions, I focus on mechanical principles that provide insights into the fundamental demands that might be made of muscle. The implications of various aspects of posture can be considered without resorting to highly detailed and constrainingly case-specific musculoskeletal modeling if we begin by assuming that:

1. costs are dominated by the demands in terms of mechanical work for supporting body weight during translation—the work associated with lifting and accelerating the limbs is not considered;
2. compression and shear can be opposed without metabolic cost using bone;
3. tension can be opposed without physiological cost through passive structures, such that muscle costs are only those associated with work and power, and not isometric tension, and
4. rigid bones can be connected by frictionless “revolute” (“hinge” or “pin”) joints.

It should be acknowledged at the outset that each of these assumptions is imperfect. In particular, a limb capable of many tasks may not be able to provide tension links with only passive tissues, and loading of muscles—even if they are not required to perform mechanical work—may impose a physiological cost. To begin with, however, we follow the assumptions outlined above in assuming that passive structures are available as long as no mechanical work or power is required.

## Scope

The purpose of this article is to demonstrate the theoretical possibility of zero limb-work gaits and to describe simple linkages with which these might be achieved, some of which might be approximated in biology. While the linkages are sometimes described in relation to specific animal forms, it is not my intention to quantify how closely species match either zero limb-work gaits or any particular linkage. Further, some linkages are described in order to demonstrate a physical principle, but that also depart from any sensible biological analogue. This need not make the demonstrated principles invalid or irrelevant, but closer anatomical analogies can generally be perceived in linkages that approach but cannot exactly match the physical limiting extremes. Two limb models are presented that have no biological counterpart, but are valuable in demonstrating the extreme point that there is no fundamental geometric reason for weight support with legs during translation of the body to demand mechanical work.

Where empirical data are presented, it is either taken directly from the literature or matches previously reported findings so closely that no novelty is claimed. However, measured force profiles will be presented to show their subjective consistency with predictions from zero limb-work gaits. It is hoped that future kinematic and kinetic studies, particularly of sprawled and crouched quadrupeds, might find value in considering their findings within the theoretical framework developed here.

## Parallels with design requirements of the early industrial revolution

A challenge faced during the industrial revolution was to convert to and from purely rotational and translational motions with only rotational joints—ultimately between pistons and gears or wheels—but without rolling or sliding elements (precluding the slider-crank mechanism). Such “straight-line mechanisms” were desirable because durable rotational joints could be machined with precision, while rolling or sliding required flat surfaces (difficult to machine before the invention of the planar (Norton 1992)) and induced wear (pending advances in materials), rapidly degrading the transmission between translational and rotational forces and motions.

The demands faced by 18th- to 19th-century engineers have a parallel in animal limbs: joints can allow effective, low-friction rotation; and work and power demands could be zero if these resulted in purely horizontal translations of the hips and shoulders without imposing horizontal forces, making effective conversion between rotations (about functional ankles) and linear translation (of hips and shoulders) advantageous. Again, animals appear limited in their use of economical rolling or sliding—they do not have wheels or skates.

## Watt’s straight-line linkage

**Fig. 1:**
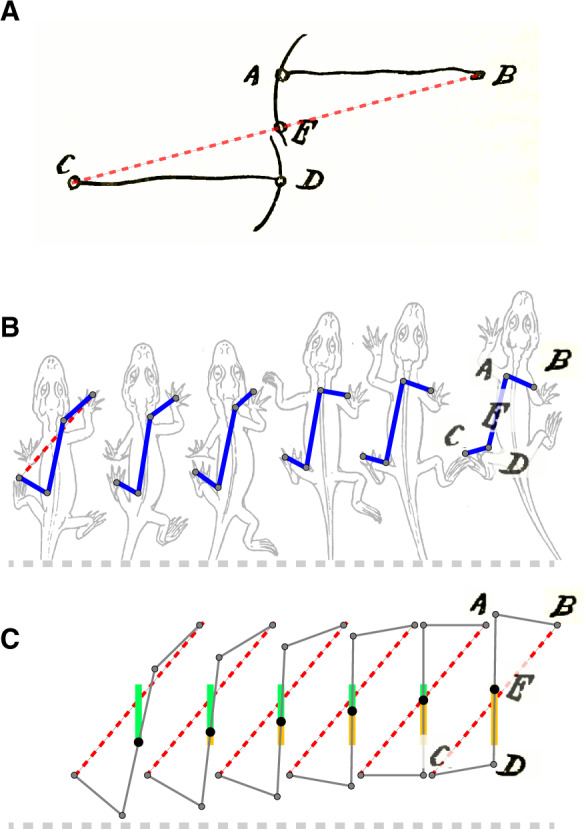
Watt’s linkage (**A**) provides an analogue for the sprawled crocodilian (Reuleaux 1876), (**B**) with diagonal foot contacts (Schaeffer 1941), (*C* and *B*) connected by a back (from *A* to *D*), forming a four-bar linkage when the ground between contact feet (red dotted line, or *C* to *B*) is included. Motion of the linkage results in the midpoint of the back (*E*) progressing (C) along a very nearly straight line (green ahead, yellow line behind). The geometry of motion of this linkage is easily calculated but not necessarily intuitive; for instance, the angular deflections *BAE* and *EDC* are not equal.

“Watt’s straight-line linkage” (Reuleaux 1876; Norton 1992) (note that Watt’s name is connected to more than one linkage) is a special case of a four-bar linkage (Fig. 1A) that provides a simple means of interchanging purely rotational motions of pin joints to very nearly linear translation. It was described by Watt for the purpose of transforming the motions provided by pistons—which need to be kept linear in order to maintain a good connection with the seals—to the rotations of a “working beam.” It continues to be widely adopted in suspension systems of cars and trains.

Many sprawled amphibians and reptiles walk with foot placements supporting weight with diagonal pairs (Schaeffer 1941; Usherwood and Self Davies 2017) (Fig. 1B)—approximately the same phasing as a trot: left hind and right fore limbs support weight and retract as right hind and left fore swing forward in protraction, and vice versa. This action can be viewed as the motions of alternating Watt’s linkages, with the links formed from: a hind limb with foot in contact with ground; a back linking hip and diagonal shoulder; the diagonal forelimb (also with a foot on the ground); and with the fourth link provided by the ground connecting the two diagonal feet.

An ideal Watt-linkage quadruped would require minimal craniocaudal (fore-aft horizontal) forces and very nearly zero limb work if roll can be neglected.

## Derivation of force profiles for near zero-work gaits approximating Watt’s linkage

If we assume a duty factor of ½ (each foot spends exactly half the stride cycle in contact with the ground) and trotting limb phasing (simultaneous diagonal fore and hind foot contact), neglecting roll or ground reaction forces other than from the limbs (i.e., weight support or drag from a trailing tail), the limb forces resulting in zero limb work—achievable for a sprawled animal using something akin to Watt’s linkage as a mechanism—can be calculated.

Watt’s linkage results in zero work if there are zero fluctuations in height or velocity and so continuous, constant weight support *mg*. Resolving vertical reaction forces *F*_z_ from hind and forelimbs gives:

(1) $$F_{z,\;\text{fore}}+F_{z,\;\text{hind}}=mg$$

If there are also no changes in pitch, pitching moments imposed due to hind and fore limb forces must cancel:

(2) $$F_{z,\;\text{fore}}r_{\text{fore}}+F_{z,\text{hind}}r_{\text{hind}}=0$$

where *r*_fore_ and *r*_hind_ give the moment arms about the center of mass of vertically orientated forces from fore and hind feet, respectively.

These moment arms change through stance, with front foot progressing toward the center of mass (decreasing the magnitude of *r*_fore_) contrasting with the hind foot translating further from the center of mass. Taking nose-up pitching as positive,

(3) $$r_{\text{fore}}=\left(1-p\right)L_{\text{back}}+\frac{L_{\text{stance}}}{2}-L_{\text{stance}}\tilde{t}$$

where *L*_back_ is the back length, defined as the distance between front and hind foot placement, with the center of mass located at a proportion *p* between hind and front limbs. *p* is determined from measured vertical impulses and the ratio of weight supported by forelimbs as a proportion of total supported weight:

(4) $$p=\frac{\int F_{z,\;\text{fore}}dt}{\int F_{z,\;\text{fore}}dt+\int F_{z,\;\text{hind}}dt}.$$

and which may also be approximated through anatomical measurements.

Stance length, *L*_stance_, is the distance traveled during one stance—assumed to be the same for front and hind limbs. $\tilde{t}\;$is the proportion of stance duration.

Contrasting with *r*_fore_,

(5) $$r_{\text{hind}}=-\left(pL_{\text{back}}-\frac{L_{\text{stance}}}{2}+L_{\text{stance}}\tilde{t}\right).$$

Combining Equations (1)–(4) gives the force profiles of fore and hind limbs through stance:

(6) $$F_{z,\;\text{fore}}=\frac{1}{2}mg\left(2p-\frac{L_{\text{stance}}}{L_{\text{back}}}+2\frac{L_{\text{stance}}}{L_{\text{back}}}\tilde{t}\right);$$

 

(7) $$F_{z,\;\text{hind}}=mg-\mathrm{mgp}+\frac{{\mathrm{mgL}}_{\text{stance}}}{2L_{\text{back}}}-mg\frac{L_{\text{stance}}}{L_{\text{back}}}\tilde{t}.$$

**Fig. 2:**
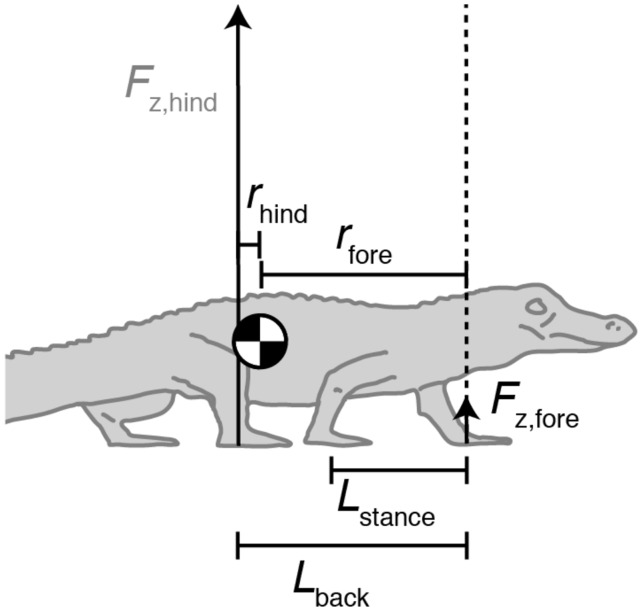
Key geometries for deriving limb force profiles for a sprawled animal approximating a Watt’s linkage as applied to an Alligator (sketch derived from Willey et al. (2004)).

**Fig. 3:**
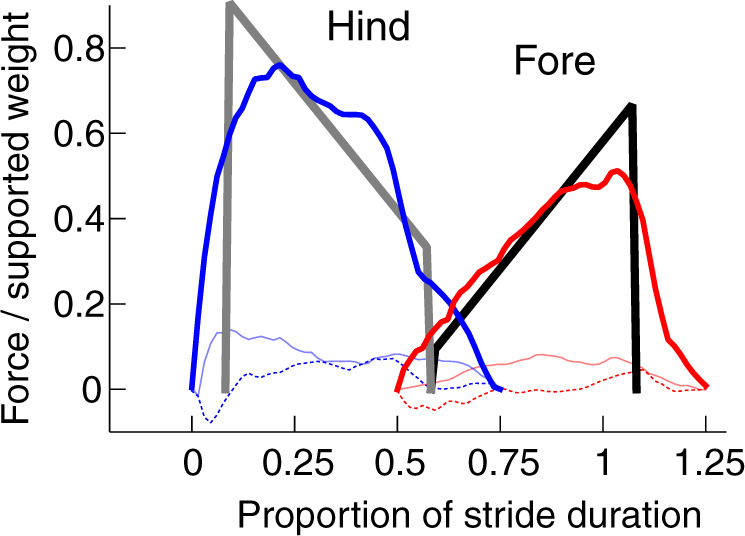
Modeled (hind, gray lines; fore, black) and measured forces of a walking alligator (Willey et al. 2004). Forces modeled assuming a near zero-work gait, potentially facilitated by motions approximating a Watt’s linkage with vertical-axis rotations of hip and shoulder. Model and empirical force traces are aligned by centroid of total vertical force.

This development allows model force profiles to be calculated given only a single side-view image (as in Fig. 2), weight *mg*, and weight distribution *p*. Force profiles from these expressions provide a reasonable match with those reported for a walking alligator (Fig. 3) in as much as the horizontal forces are relatively low and the fore forces are skewed high in late stance, while hind forces are skewed high in early stance.

Note that these skews are not an inevitable consequence of having forelimbs progressing toward the center of mass, and hindlimbs traveling away. Were the weight-supporting feet to average directly under their respective shoulders and hips, and the forces to be orientated through these proximal joints—as generally assumed in vaulting or bouncing idealizations—vertical force profiles would be symmetrical and there would be large deceleration–acceleration craniocaudal forces. Skewed forces are the result of resolving pitching moments at the same time as more-vertical-than-purely-axial forces.

## The principle revealed from Watt’s linkage

**Fig. 4:**
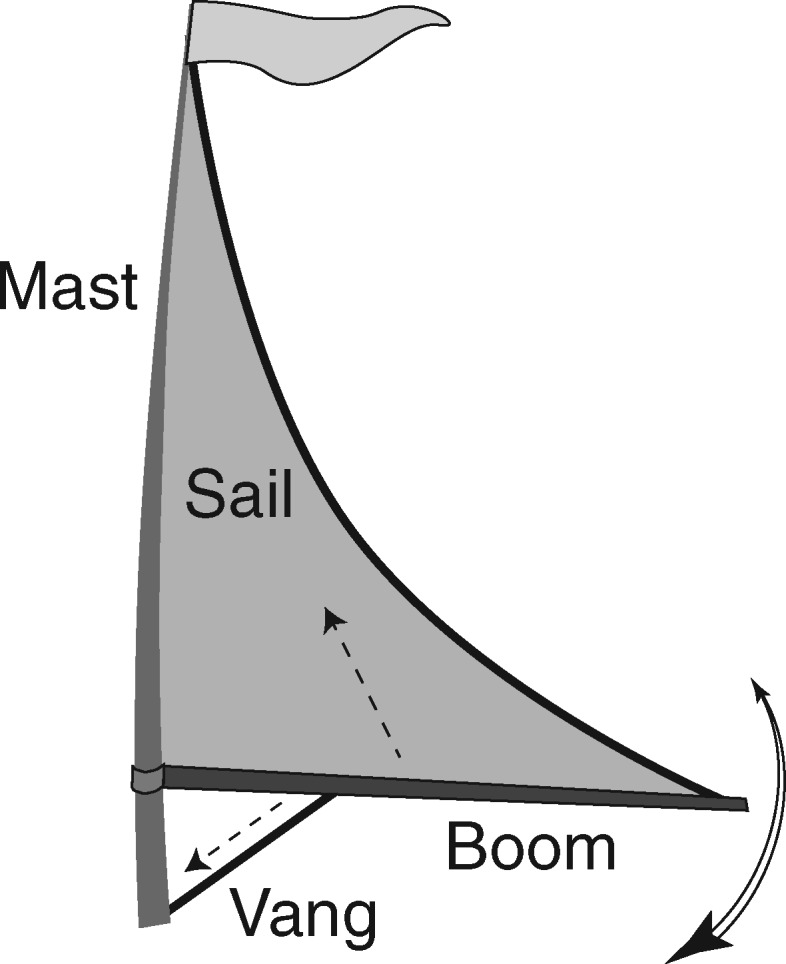
The boom of a sailing boat demonstrates that vertical forces can be opposed with passive structures while allowing free horizontal rotations about a vertical-axis joint. The boom is free to rotate about the mast (double headed arrow) while loaded in compression; vertical forces are supported with tension (dashed arrows) upward (sail) and downward (boom vang or kicking strap).

Watt’s linkage allows weight to be supported during translation of the center of mass without demanding mechanical work from the limbs. This requires that rotations about joints are achieved without torques about the same axis. The sprawled posture enables this, as the axis of rotation of hips and shoulders approximates vertical—the direction of weight support—and forward motion is horizontal. The analogy of the boom of a sailing boat demonstrates that this could feasibly be achieved with biological structures without unrealistic mechanisms: the boom, analogous to femur or humerus bones, resists compression and achieves low-resistance rotations about the mast supported by tension along the sail (up) or boom vang (down) (Fig. 4), analogous to passive tensile tissues acting across shoulder or hip. The general principle is simple: horizontal translation need not demand mechanical work if weight can be supported with frictionless vertical-axis joints.

## But no animal is a perfect Watt’s linkage…

A quadruped with rigid links forming Watt’s linkage, especially with relatively short limbs and long back, would require fluctuations in energy for a non-point-mass body: the back yaws, so fluctuations in rotational energies would require cycling of work. Further, to varied extents, animals bend their backs and have mass centers located other than centrally between hips and shoulders. An expectation of true zero limb work in amphibians, lizards, and crocodilians might be foolish, and analogy with Watt’s linkage should be made with caution. However, while Watt’s linkage is the simplest that can be conceived for effective quadrupedal locomotion with near-zero limb work, this does not prevent more complex linkages from achieving the same—or slightly better—lack of resistance to horizontal travel. Does lateral flexion of the back necessarily mean mechanical work demand? No, not if the flexion can be achieved with rotations of vertebrae with vertical axes. This principle is exploited in some toy snakes.

## The screen support and the tortoise: the potential for true zero limb work

**Fig. 5:**
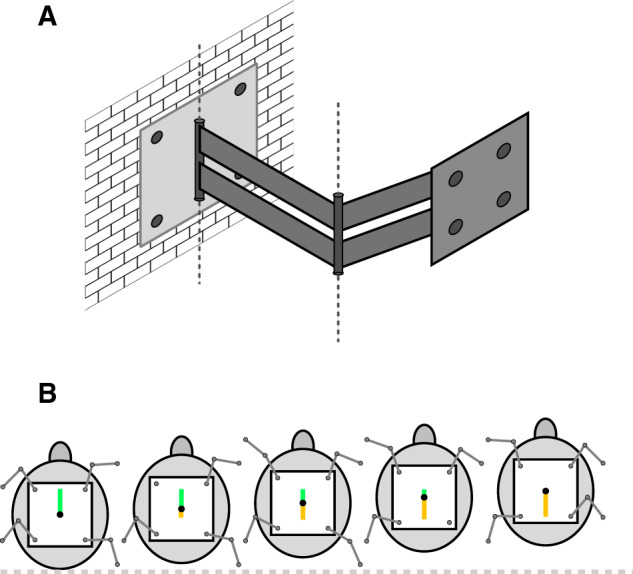
The tortoise modeled as having two vertical-axis joints per limb, as in a pull-out monitor screen (**A**), allows not only exactly straight-line, horizontal, zero-work motion of the center of mass (green line ahead, yellow behind, (**B**)), but can also continuously prevent both pitching and rolling moments.

Monitor screens are often mounted to walls with a two-link mechanism enabling free translation of the screen while providing weight support (Fig. 5A). Again, this linkage divorces weight support from motions with vertical-axis joints and horizontal movements. This two-joint mechanism can be viewed as an analogy for a sprawled limb with not only vertical hip (or shoulder) joint, but also a vertical-axis knee (or elbow) joint. This is definitely not intended to be an accurate reduction for tortoise limb action, which certainly does include a degree of rotation, particularly of the distal limb segments, in parasagittal planes (Schmidt et al. 2016). However, the principle provides an additional degree of freedom (per limb) which means that horizontal translation can be continuously supported without horizontal force or fluctuations in height.

**Fig. 6:**
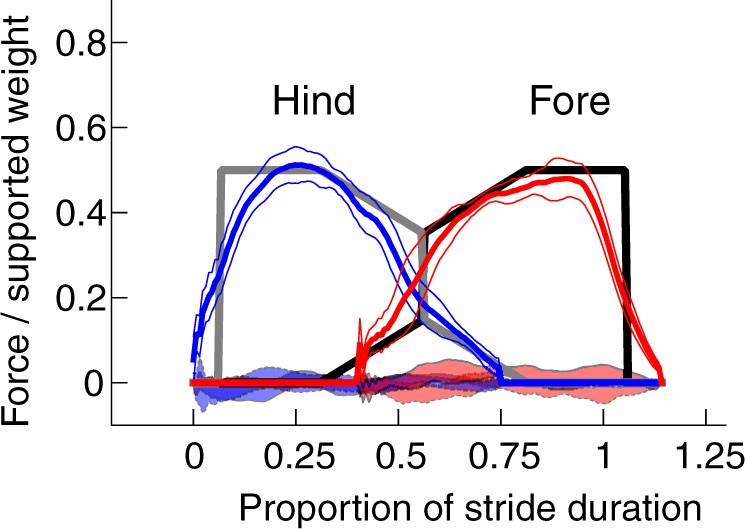
Measured (bold colored lines bounded by +/−SD) and modeled force profiles for a walking tortoise. Model and empirical force traces are aligned by centroid of total vertical force. The model is for a tortoise that supports continuous, straight-line motion of the center of mass without pitching or rolling following Jayes and Alexander (1980). In this model, which has exactly three limbs supporting weight throughout the gait cycle, rapid transitions in force are required, which would be physiologically unrealistic (as discussed in Jayes and Alexander (1980)). However, this is not a necessary feature of the zero limb-work strategy; if periods of three-limbed support were interspersed with periods of four-limbed support, smooth transitions in force would be possible. However, the system then becomes overdetermined for periods of the gait cycle, making prediction of forces at these times with the current model impossible. Modeled vertical force profiles (gray hind; black fore) and zero horizontal forces would result in zero mechanical work demand from the limbs in supporting body weight during translation. Measured vertical forces broadly match modeled (*N *=* *10 (hind) and 8 (fore) stances), and horizontal forces (shaded color regions) are low, agreeing with Jayes and Alexander (1980).

Center of mass translation could be achieved with zero limb work throughout stance; further, rotations (in roll, pitch, or yaw) could be completely avoided given a suitable footfall pattern (Gray 1944; McGhee and Frank 1968; Jayes and Alexander 1980). A model for tortoise forces was developed by Jayes and Alexander (1980) (equivalent to the derivation above but also resolving roll), originally to explore the requirements of avoiding excessive pitch and roll deflections in very slow gaits. The same assumptions and model (so not repeated here) resulting in zero roll, pitch, horizontal or vertical acceleration also results in a zero limb-work—not merely work minimizing—gait. Tortoises approximate this mechanical analogy: giant Galapagos tortoises have been measured achieving near-constant weight support overall (Zani et al. 2005); individual limb vertical forces (Jayes and Alexander 1980; Fig. 6; see Supplementary Movie 1) approximate those predicted to provide continuous weight support without rolling or pitching (Jayes and Alexander 1980); and limb horizontal forces can be low. Limb forces may impose considerable moments about hip and shoulder joints, but do not demand mechanical power as long as both forces and joint axes are vertical.

The sprawled posture and walking gait of tortoises should not, therefore, be considered in some way mechanically deficient because they lack “pendular transduction” between kinetic and potential energies. Such interchange provides no advantage in the hypothetical extreme of absolute zero limb work.

It should be highlighted that, while a sprawled posture indicates that at least some joints within the limbs are orientated at least some way toward vertical, the tortoise leg is certainly not composed of only purely vertical joints. The screen-support mechanism is intended to demonstrate a potential physical extreme and a feasible realization of this extreme—that zero limb work progression is possible with vertical-axis joints. The consistency between zero-work force profiles and measured tortoise limb forces is encouraging; however, no quantification is attempted here concerning the relative contributions of vertical-axis joints and other potential straight-line mechanisms.

## What of the parasagittal limb?

The parasagittal limb of most mammals and many dinosaurs contrasts with sprawled limbs in that they operate predominantly in the forward-back, up-down plane. As a result, their joints must rotate about lateral axes, and the principles of economical weight support demonstrated by Watt’s linkage cannot be exploited. Does this mean that the parasagittal limb is inherently unable to provide purely vertical forces throughout stance and the zero limb-work in stance condition approximated by tortoises? Theoretically at least, no.

## The Peaucellier linkage: a hypothetical zero limb work parasagittal limb

**Fig. 7:**
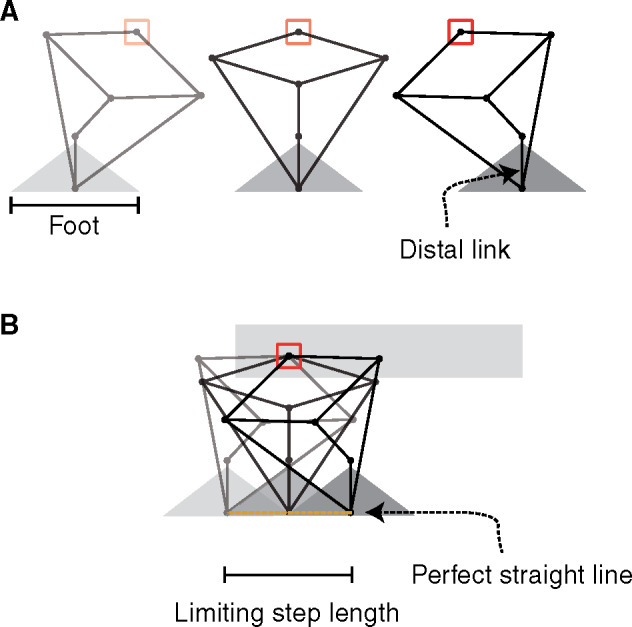
The Peaucellier linkage demonstrates that perfectly straight-line motion can be achieved with a parasagittal limb consisting of only lateral-axis joints; weight could be supported during steady, horizontal translation without requiring work. Three postures in a sequence are shown (**A**), and combined in a body-fixed frame of reference (**B**). The red boxes provide a fixed body-reference for comparison between postures and presentations. The joint within the red box—the functional hip or shoulder—is free to rotate. In addition to no horizontal forces or fluctuation in vertical force during translation, no moments are applied to the body; the red box does not rotate. The linkage is not similar to any limb described in nature and is limited to providing weight support when the proximal joint is above the supporting “foot.” Beyond this, the most distal link is not maintained vertically, and the linkage collapses.

Many linkages have been described and implemented that achieve parasagittal limb motions using lateral-axis joints that support weight with near-horizontal motions of the functional “hip” or “shoulder,” maintaining also low horizontal forces, resulting in low mechanical work demands. A full survey of these linkages falls beyond the scope of this article; however, the Chebyshev “Plantigrade machine” displayed in Paris in 1878 and the more modern “Strandbeest” of Jansen deserve particular note (and are effective internet search terms). The Peaucellier linkage (Fig. 7) demonstrates that the extreme—of perfect horizontal translation, zero limb work weight support during translation—is theoretically achievable using parasagittal limbs.

However, this linkage is not typically adopted in walking machines. It is limited in practical utility as a leg because the bottom link must be kept vertical; and this is only achieved (without a foot that actually grasps the substrate) if the force vector passes through the foot; step length is limited to foot length. It also deviates sufficiently from animal form that, even if this exact linkage did offer benefits, this limb design might not be achievable by any reasonable evolutionary process. The point still stands, however: parasagittal limbs with lateral-axis joints could require exactly zero limb work associated with weight support during travel.

## A biorealistic four-bar linkage could achieve near-zero-work weight support

**Fig. 8:**
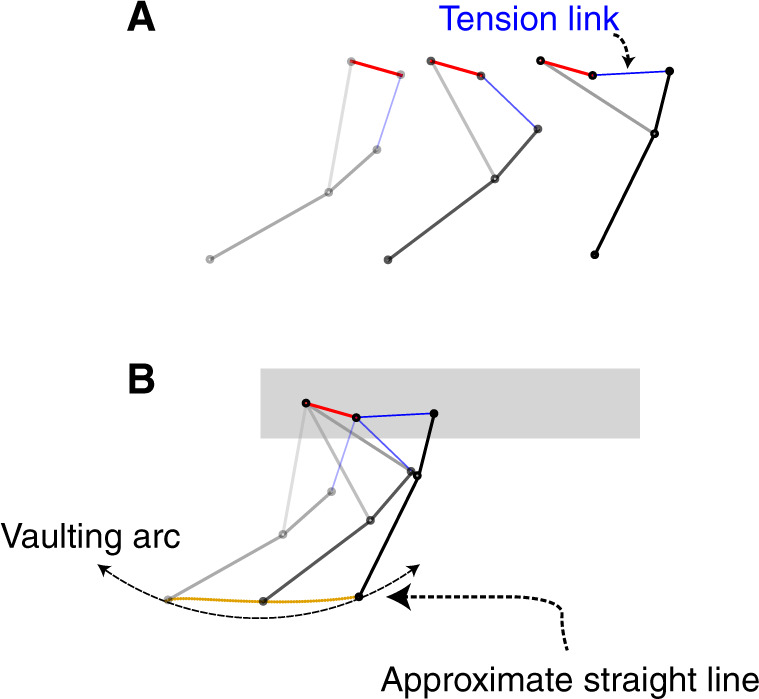
An appropriately tuned four-bar linkage allows very nearly straight-line motion with lateral-axis joints resulting in a parasagittal limb. Three postures in a sequence are shown (**A**), and combined in a body-fixed frame of reference (**B**). This linkage produces near-horizontal motions between foot and hip or shoulder, resulting in very low work demand during steady translation supporting body weight. The red link provides a body-fixed reference: joints at each end are free to rotate, but this link is fixed to the body. The linkage could be viewed as broadly biorealistic, with a zero-length-change tension element (blue) that could represent a completely passive ligament or low-cost, isometric muscle-tendon unit. The triceps (forelimb) and quadriceps (hindlimb) could approximate this role in the upper segments of upright parasagittal mammal legs, though other joints, structures, and indeed linkages of greater complexity could all contribute toward generating a straight-line mechanism with low limb work demand.

No simple four-bar linkage results in a perfectly straight-line mechanism; however, many possibilities exist that get very close (Norton 1992). Approximately straight-line horizontal motion supporting very nearly pure vertical forces—and so almost zero-work weight support during stance—could be achieved with a broadly biorealistic four-bar linkage with appropriately tuned dimensions (Fig. 8; Supplementary Movie 2).

Low-fidelity prototypes of four-limbed models (Supplementary Movies 3, 4) using the tuned four-bar linkage show how weight can be supported continuously with a footfall pattern providing a continuous base of support (each limb contacting the sub/superstrate for three-fourth of the stride cycle, with forelimb contacting one-fourth the cycle after the hindlimb on the same side). These models support the prediction: the body need not rise or fall during stance, and the limbs oppose only those horizontal forces associated with joint friction.

Is such a strategy perfectly adopted by parasagittal quadrupeds such as mammals? Certainly not: most large mammals walk with a duty factor (0.6–0.7, Usherwood and Self Davies 2017) that is insufficient to meet the requirements of continuous weight support without rolling and pitching (≥ 0.75, Jayes and Alexander 1980); fore-aft forces are sometimes consistent with approximately axial limb loads (Alexander 1989) as are energy fluctuations (e.g., Cavagna et al. 1977); and some degree of stiff-limbed vaulting is broadly consistent with somewhat M-shaped vertical forces typical of moderate to large walking mammals, and footfall timing during grazing (Usherwood and Smith 2018). However, while perfect zero-work walking is certainly not adopted by large walking mammals, the hypothetical extreme case presented here may provide a useful concept when considering function and diversity of two-joint muscles. For instance, do some species approach zero-work strategies more closely than others?

## Discussion

**Fig. 9:**
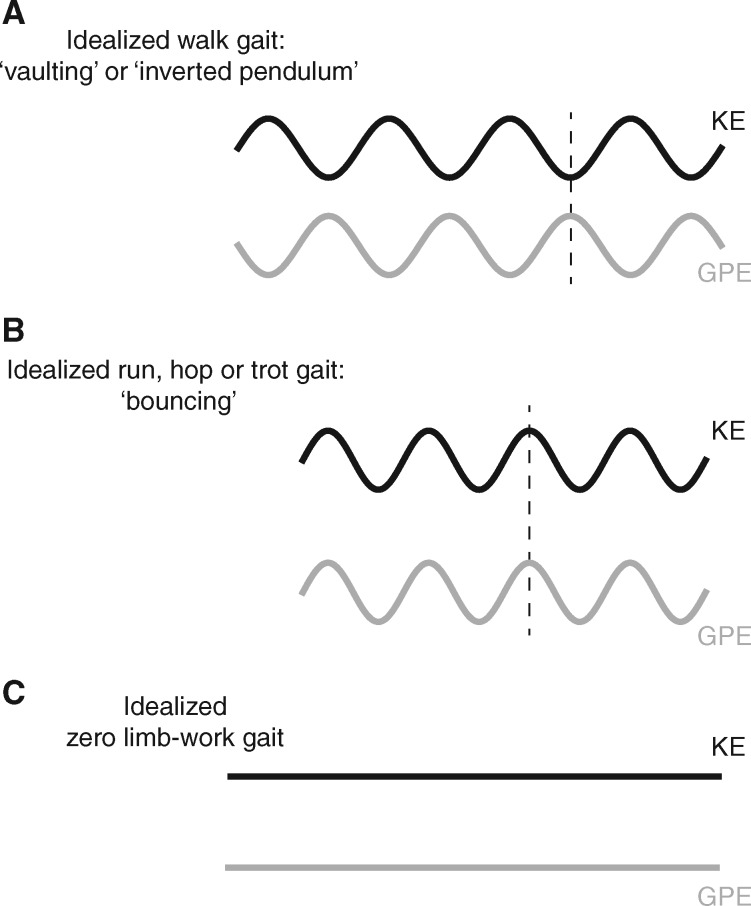
“Kinetic” definitions of gaits based on energy fluctuations through the time of (following Cavagna et al. (1977) and Biewener (2003)): walking, vaulting or “inverted pendulum gaits” (**A**); running, hopping, trotting, or “bouncing gaits” (**B**); and the proposed “zero limb-work gait” available to quadrupeds, or bipeds with very large pitch moments of inertia (**C**). The “inverted pendulum” mechanism involves out of phase fluctuations of kinetic and gravitational potential energy; when the body is high (dashed lines), with high gravitational potential energy GPE, it is also slow, with low kinetic energy KE. This involves relatively low limb work as forces and velocities are maintained broadly perpendicular to each other—as in a pendulum. The “bouncing” gaits show in-phase fluctuations of kinetic and gravitational potential energy (when GPE is high, so is KE) and so relatively large fluctuations of mechanical energy, associated with forces and velocities deviating from perpendicular. Some energy may be returned each step through elastic recoil. The “zero limb-work gait,” when ideal, shows zero fluctuation in kinetic or gravitational potential energy—just as would be the case for a vehicle rolling on circular wheels.

The success of work-minimizing accounts for walking and running in point-mass models of bipeds (Alexander 1980; Srinivasan and Ruina 2006) is appealing ... or perhaps beguiling. Some form of minimization of mechanical work has also been invoked as an account for quadrupedal gaits (Alexander 1980; Ruina et al. 2005; Srinivasan and Ruina 2006; Xi et al. 2015; Polet and Bertram 2019; Usherwood 2019). However, quadrupedal gaits could, provided appropriate straight-line mechanisms were anatomically realizable, require consistently zero power, at least in terms of the limb mechanical work associated with weight support during steady translation. Vaulting in quadrupedal walking should therefore NOT be viewed as a strategy for maximizing mechanical economy using direct analogy with bipedal work minimization, unless near-axial loading is already accepted as a given for some reason (Alexander 1980; Xi et al. 2015; Polet and Bertram 2019). Even in bipeds, a finite pitch moment of inertia and non-axially loaded legs reduce mechanical work demands in running (Usherwood and Hubel 2012). Alexander identified the potential energetic advantage of some form of linkage for quadrupeds, formed of links consisting of bones and muscle or passive tension tissues (Alexander 1976, 1988, 1991), including the potential of two-joint muscles and pantograph mechanisms, but stated “mammals seem not to have adopted this [these] solution[s]” (Alexander 1991). He therefore focused his modeling of large parasagittal locomotion on strategies for work minimization given the assumption of axially loaded limbs. The case of the tortoise, however, demonstrates that near-zero stance-work limbs are sometimes realized in biology; and it may be worth revisiting the role of two-joint muscles and four-bar linkages in small, flexed-limb mammals exhibiting slow, level locomotion. The zero limb-work strategy provides a good account for the vertical force profiles and (absence of) horizontal forces in alligator and tortoise (Figs. 3 and 6) and offers a useful fundamental mechanical paradigm in addition to those of pendular vaulting and spring-like “bouncing” gaits described for parasagittal bipeds and quadrupeds (Cavagna et al. 1977). When expressed in terms of energy fluctuations, as has become the convention when describing “kinetic” definitions for gaits, the zero-work paradigm is easily identified (Fig. 9). The walking, “inverted pendulum” or “vaulting” gait described for slow bipeds and large, parasagittal quadrupeds (since Cavagna et al. (1977)) is characterized by out-of-phase fluctuations in kinetic and gravitational potential energy (KE and GPE respectively) and generally low limb work because forces are broadly perpendicular to velocities during the vaulting phase. The “bouncing” gait of running, hopping or trotting shows in-phase fluctuations of kinetic and gravitational potential energy (see Cavagna et al. (1977) and Biewener (2003) for a current text-book presentation) with relatively high limb works due to forces and velocities deviating from perpendicular. Some degree of recoil from elastic structures prevents all the positive limb work from being demanded *de novo* each step. The zero-work gait would be characterized by zero fluctuations in kinetic or gravitational potential energy, just as would be observed for a rolling wheel or round ball.

The straight-line mechanisms described here demonstrate the theoretical potential for such gaits to be achieved with passive structures, without the physiologically cost of activating muscle to oppose weight-induced forces or for simultaneous positive and negative power generation/dissipation.

Why do large mammalian quadrupeds not adopt zero limb-work gaits? Limbs adapted for high speed locomotion, especially noting the costs associated with protraction (a requirement for legged locomotion but not wheeled) and advantages due to axial limb loading, might well invalidate some of the assumptions in this article. Whether this is necessarily the case among mammals, especially small, slow, and flexed-limbed quadrupeds, deserves further attention.

## Practicalities 1. What differentiates a “zero-work gait”? How do I know if an animal is (nearly) adopting one?

The GPE–KE idealization for distinguishing gaits (Fig. 9) may serve to demonstrate the principles behind distinguishing between the three “gait paradigms,” but the reality may often be gray. No gait results in an absolutely zero fluctuation in KE and GPE; when is “nearly zero” near enough to indicate approximation to the zero limb-work gait? One approach to this is to declare how much fluctuation would be consistent with the alternative gait paradigms. For vaulting gaits, this is simple to calculate for parasagittal animals assuming stiff-limbed geometries. At low walking speeds, potential energy fluctuations as a proportion of those predicted from vaulting—termed the Compression Ratio (Usherwood et al. 2007)—in dogs is approximately 60%. At moderate to high walking speeds, this falls to approximately 20%. Might this imply that the “vaulting” gait paradigm transitions toward the “zero limb-work” paradigm with speed? How this criterion might be applied to sprawled animals is more problematic: how much “should” a tortoise go up and down if it was using a vaulting gait? But that also highlights a point being made here: perhaps it is obvious that a sprawled animal “shouldn’t” go up and down much ... but there again, another way of putting that is to say “why wouldn’t a sprawled animal be using a zero limb-work gait?”

Limb powers can be calculated from measured single-limb forces and center of mass motions. An animal approximating a zero limb-work strategy would display approximately zero limb powers. The challenge with this is that, unlike the energy fluctuation approach, it is less immediately apparent what power profiles would be consistent with the alternative gait paradigms; this may be solved in the future. But without these comparisons with which to compare the measured limb powers, it will be difficult to assert that a power is sufficiently near zero to be interpreted as indicating adoption of the zero limb-work strategy.

Force profiles offer a better indicator that zero limb-work strategies are being adopted—hence the approach taken here. At least in their simplest renditions, neither pendular “vaulting” nor spring-like “bouncing” gaits would predict skews in vertical ground reaction force; and both would result in axial limb forces resulting in large horizontal forces.

So, when a gait is measured that meets the following criteria, perhaps we should infer that the zero limb-work gait strategy may be being approached:

1. for parasagittal animals, a low compression ratio (a relatively small fluctuation in height with respect to stiff-limbed vaulting);
2. an early skew in hindlimb vertical force;
3. a late skew in forelimb vertical force; and
4. low horizontal forces, such that forces are orientated more vertically than through proximal (hip or shoulder) joint.

That many of these criteria may also be met in animals adopting gaits currently described as vaulting or bouncing gaits suggests that these gait distinctions are not all-or-nothing; there may be gradual transitions between gait strategies, and a gait may be nearly-but-not-quite zero limb-work and may also display vaulting or bouncing kinetics.

## Practicalities 2. How do I know if a structure, mechanism, or tissue is facilitating a (nearly) zero limb-work gait?

While I have demonstrated that it is possible for vertical-axis limb joints in sprawled limbs, and linkages in parasagittal limbs, to result in zero limb-work weight support during translation, I have not demonstrated that this is actually the case in any system. Where should we be looking and how do we know if we have found such a structure, mechanism, or tissue? The principle is simple: a mechanism that allows horizontal motions without horizontal forces can facilitate the zero limb-work strategy. Vertical-axis joints do this perfectly, but near-vertical would also be pretty effective.

I have shown one potential four-bar linkage that would enable near-zero limb-work; however, many others exist, and there is no reason for biological limbs to be limited to only four bars. When might a link or linkage be interpreted as functioning to enable a near-zero limb-work strategy in a parasagittal limb? First, the linkage must be flexed at midstance: in order for zero vertical motions, the leg must be more compressed at midstance than at the extremes of stance. Second, each link of the linkage must be isometric: each supports a load without deflecting and all avoid either negative or positive work. Third, the range of positions accessible to the linkage must be broadly horizontal.

If we are interested in exploring the tissues that enable low-work translation during weight support, we presumably accept bone to be rigid, and should focus on muscle-tendon units that do not change length, and so do not perform work. The muscles involved may therefore be relatively small. They may also change depending on context. Nevertheless, they may prove to be vital for facilitating economical locomotion.

## Acknowledgement

This work has been improved through discussion with members of the Structure and Motion lab, reviewers and editors, and the wider biomechanics community. The abstract translations were kindly provided by: Jialei Song, Ulrike Muller, Masateru Maeda, Romain Pintore, Grzegorz Sobota and friends. I am grateful to the owners of various tortoises for their time and advice; ‘Torchie’ is featured here. Tortoise measurements made following informed consent of owners and with RVC ethical approval URN 2018 1780-3.

## Funding

This work was supported by a Wellcome Trust Fellowship 202854/Z/16/Z.

## Supplementary data

Supplementary data available at *IOB* online.

## Supplementary Material

obaa017_Supplementary_Data
